# Remineralization potential of apacider gel on enamel and cementum surrounding margin of ceramic restoration

**DOI:** 10.4317/jced.61629

**Published:** 2024-07-01

**Authors:** Niwut Juntavee, Apa Juntavee, Chansin Khunngoen, Akarin Singpromma, Warun Khoosuwan

**Affiliations:** 1 Department of Prosthodontics, Faculty of Dentistry, Khon Kaen University, Khon Kaen, Thailand; 2 Division of Pediatric Dentistry, Department of Preventive Dentistry, Faculty of Dentistry, Khon Kaen University, Khon Kaen, Thailand; 3 Division of Biomaterials and Pediatric, Faculty of Dentistry, Khon Kaen University, and Dontan Hospital, Thailand

## Abstract

**Background:**

Modern management of carious lesions has been targeted upon using remineralizing agents. This study investigated the remineralization potential of apacider gel (AG) and casein phosphopeptide-amorphous calcium fluoride phosphate (CA) on enamel and cementum around the cavosurface area of the ceramic margin.

**Material and Methods:**

Seventy-five extracted human mandibular molars were sectioned at 0.8 mm above and below the cemento-enamel junction (CEJ) to remove the CEJ portions and replaced them with glass ceramic disks by bonding them to the crown and root portions with resin cement. The enamel and cementum area of 4x4 mm2 surrounding ceramic was demineralized with Carbopol–907. The demineralized surfaces were treated with either AG or CA, while one group was left with no treatment (NT) and served as control. Vickers microhardness was determined before-, after demineralization, and after remineralization. The percentage of hardness recovery (%HR), and remineralization potential (%RP) were analyzed with ANOVA and Bonferroni’s test (α=0.05). Polarized light microscopy (PLM) was assessed for lesion depth. Scanning electron microscopy (SEM) was investigated for surface alterations.

**Results:**

Significant differences in remineralization were found upon various remineralizing agents compared to NT for both enamel and cementum (*p*<0.05). No significant difference in %HR and %RP was observed between AG and CA (*p*>0.05). However, AG signified greater decrease in lesion depth and better improvement in surface characteristics for both enamel and cementum than CA.

**Conclusions:**

AG possesses comparable remineralization ability to CA. However, decreasing in carious lesion depth was evinced with using AG more than CA. AG was recommended as a potential remineralization material for handling initial caries for both enamel and cementum.

** Key words:**Apacider, artificial carious lesion, CCP-ACFP, remineralizationCare Team.

## Introduction

Considerably increasing demand in esthetic dentistry has directed several newly developed ceramic restorative materials for clinicians to provide extremely aesthetic restorative treatment with the simplicity of construction through digital dentistry. Still, the accuracy of restoration fabricated from all-ceramic is quite negotiated in contrast to ceramic veneered metal. Inadequate adaptation of the restorative margin to the prepared abutment tooth convinces bacterial plaque accumulation, which initiates dental caries near the restorative margin, commonly located either on cementum or enamel, adjacent to the cemento-enamel junction (CEJ) in advanced fixed prosthodontics reconstruction (1). This is also associated with the prevalence of root caries in geriatric persons (2,3). Dental caries surrounding the margin of restoration were considered a major cause of failure that restricts the restoration longevity (4). Critically, dental caries beneath a margin of the restoration are barely noticeable by radiograph (5).

Dental caries exist as a consequence of biological processes of bacteria attacking tooth structure, which specifically depends on the host diet (6,7). The fundamental mechanism of dental caries is founded on the demineralization of tooth structure from the acid attack produced by bacteria, mainly *Streptococcus mutans* and *Lactobacillus*, that colonize in dental plaque biofilms (8). The demineralization phenomenon is a consequence of the surplus rate of the calcium phosphate (Ca3 [PO4]2) dissolution over the remineralization processes owing to the disparity in ion concentration pitch amid tooth and saliva (9,10). Though, dental caries is an avoidable infectious disease, the inhibition of caries progression is limited to the demineralized-remineralized process in the incipient caries lesions as a white spots appearance which is usually located in the superficial level of a tooth (9,11). The early carious lesion denotes the most primitive indicator of tooth decay, wherein the superficial enamel surface is still continuously undamaged, whereas the beneath layer is demineralized, hence leading to dental cavitation if unappropriated care is introduced. The research has been shifting to the improvement of approaches for the detection of initial carious lesions and early administration with a non-invasive approach throughout the remineralized process to reestablish the demineralization/remineralization balance. In the initial demineralized phase, the inorganic substances are removed, but still subsequently maintained a rigorous superficial interprismatic foundation, which permits the feasibility of a reversible procedure via remineralization (12). This is capable of reestablishing a vigorous normal enamel or discontinuing carious dissemination (13). Currently, a customary approach in restorative dentistry is principally focused on a remineralization strategy to restore the tooth structure. Normally, remineralization is the process by which the calcium ions (Ca2+) and phosphate ions (PO43-) are provided from an external source to stimulate ion installation into crystalline defects of the demineralized tooth. Wide varieties of unique Ca3 [PO4]2 based products have been introduced for the remineralization process, mostly based on either crystalline or amorphous calcium phosphates aside from fluoride-based structures. The demineralization relates to the destruction of the crystalline conFigurations of hydroxyapatite (HA) at the surface and inner zone from the bacterial-produced acid (14). The remineralization naturally occurred by accomplishing the re-establishment of the impaired crystalline HA foundation. This process conveys in the physiologic situations at neutral pH wherein the PO43- and Ca2+ from saliva is resettled back to the carious area, aiding as the assembly for the vaster crystal HA that is somewhat sounder to confront with bacterial acid striking (12). Fundamentally, the process of demineralization & remineralization happens instantaneously at the tooth surface and involves a significant amount of inorganic contents on the crystalline HA (15). As the demineralization process is foremost, it breaks up the HA crystal arrangement, and then ultimately originates the dental cavity. Yet, an incompletely demineralized HA crystal foundation could be reestablished to its original phase if it is adequately subjected to the oral conditions that dominantly intensify the remineralized process, directing to the repairable situation of the caries lesion (16).

Fluoride has been used as a classical agent for dental caries prevention (17). It could inhibit the loss of inorganic materials from the tooth surface by promoting absorption toward the crystal networks along with attracting Ca2+ and PO43- ions from saliva to formulate fluorapatite. hence increasing enamel endurance from acid attacks. Nevertheless, the process of remineralization can be a limited achievement because of the obtainable quantity of Ca2+ and PO43- ions in the oral conditions. Fluoride is incapable of steering genesis in the mineral orientation of the tooth in physiologic situations, which is crucial in recovering the physiological properties of the demineralized tooth (18). The fluoride just only diminishes the demineralization rather than supporting remineralization to neutralize the inorganic impairment of the HA crystal configuration. Yet, a tremendous fluoride installation towards the surface layer of enamel perhaps prohibits the dispersion of ions getting through the inner part of caries, thus causing the incomplete remineralization process (14). This principally achieves solitary surface remineralization, with slight structural improvement of the deeper carious lesion (19,20). Several alternative materials to fluoride have been commenced based on their anti-cariogenic capabilities (11,21-23). The casein phosphopeptide–amorphous calcium phosphate (CPP-ACP) was initially introduced which potentially exhibits anti-cariogenic occurrence as well as intensifies remineralization (24). An extreme amount of Ca2+ and PO43- ions steer the ions diffusion into the carious lesion but still maintain the ion concentration gradient during the remineralization process. The CPP-ACP fairly attaches to plaque, and vastly offers the Ca2+ ions pool, slowly releasing free Ca2+ ions diffusion, and additively reduces carious lesions similarly to fluoride (25). Subsequently, the casein phosphopeptide–amorphous calcium fluoride phosphate (CPP-ACFP) was commenced in crème form for easily smeared on the surface of teeth. It potentially penetrated the deep lesion with no spontaneous precipitation, resulting in subsurface remineralization as well as fluorapatite formation in the body of the carious lesion (26). Recently, apacider gel (AG, Biomaterial research, KKU, Khon Kaen, Thailand) was introduced as a novel alternate material for the treatment of early caries lesions (22). The AG is predominantly composed of apacider-AW® (Sangi, Tokyo, Japan) that was designed to be incorporated in the form of gel to preserve the integrity of the manufactured materials. It possesses antibacterial properties that are founded on HA including zinc (Zn), silver (Ag), and calcium-phosphate with silica (27). *In vitro* studies indicated that apacider is capable of significantly increasing the microhardness of enamel and enhanced remineralization of white spot lesions comparable to fluoride varnish and CPP-ACP (22,28). The AG can enhance remineralization through Ca3[PO4]2, while the Ag2+ intensely combats against cariogenic bacteria, especially *S. mutans* (27,29). The tackiness of AG helps the material to better stick and stay on the teeth for a long time compared to other remineralizing materials. Also, this material has quite a similar color to the tooth and does not induce a yellowish color to the tooth surface (23,30). Excessive fluoride intake during tooth development can remarkably cause alterations in the tooth structure for instance stain, and dental fluorosis. Whereas the composition of AG is an antibacterial property based on Ag2+ and Zn2+ which are minimal toxicity plus enrich remineralization potential from Ca3[PO4]2. As such, this study intended to investigate the possibilities of AG compared with CCP-ACFP on the remineralization potential of artificial carious lesions neighboring the cavosurface margin of restorative ceramic. The outcomes are cogitated as new preventive means to inhibit caries initiated surrounding ceramic restoration. The null hypothesis is set as no significant difference between AG and CCP-ACFP on the capability to remineralize initial caries on enamel and cementum.

Materials and Methods

**Figure 1:**
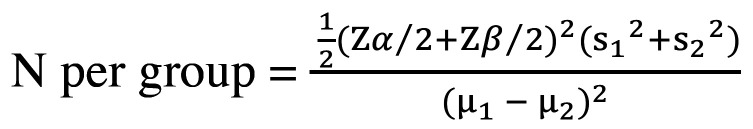
Equation 1.

This investigation was performed through the ethical exemption from the KKU research committee for ethics in human research (HE #592078) concerning the quality guidelines of in-vitro study according to the CRIS checklist. The sample size was appraised according to previous data at 95 % of testing power through Equation (1) with Piface program version 1.76 (University of Iowa, Iowa, IA, USA) (29), (Fig. 1).

Which: normal standard deviation, Zβ=1.28 (β=0.1); Zα=1.96 (α=0.05), mean, µ1=289.16; µ2=268.35, and standard deviation, s1=20.98; s2=26.49. The 25 samples per group were accomplished.

-Sample preparation

**Figure 2:**
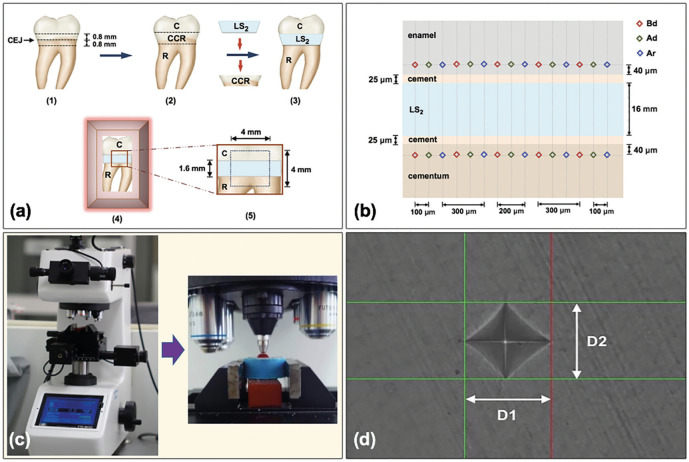
(a) The crown (C) and root (R) of the human third molar were horizontally sectioned at 0.8 mm above and below the cementoenamel junction (CEJ) (1) and replaced the C-CEJ-R (CCR) portion with lithium disilicate ceramic (LS2) by bonding to the C and R portion with resin cement (3), and then placed in an acrylic block (4) to create a flat surface of area 4×4 mm2 (5) for (b) determination of microhardness of enamel and cementum at before demineralization (Bd), after demineralization (Ad), and after remineralization (Ar) (5 locations each, 100 microns apart) at 40 microns from the junctions of resin cement to enamel and cementum with (c) diamond indented Vickers microhardness tester. The indentation (d) was measured for diagonal length (D1, D2) and calculated for Vickers hardness number.

**Figure 3:**
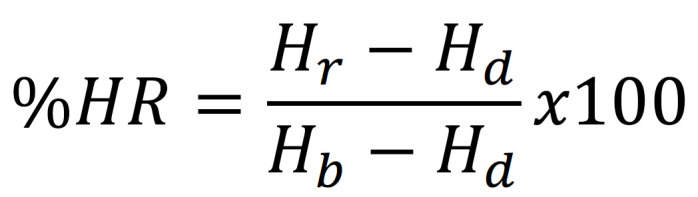
Equation 2.

**Figure 4:**
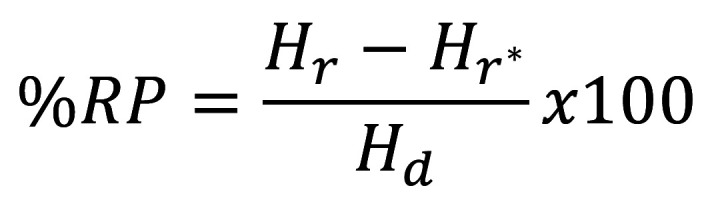
Equation 3.

**Figure 5:**
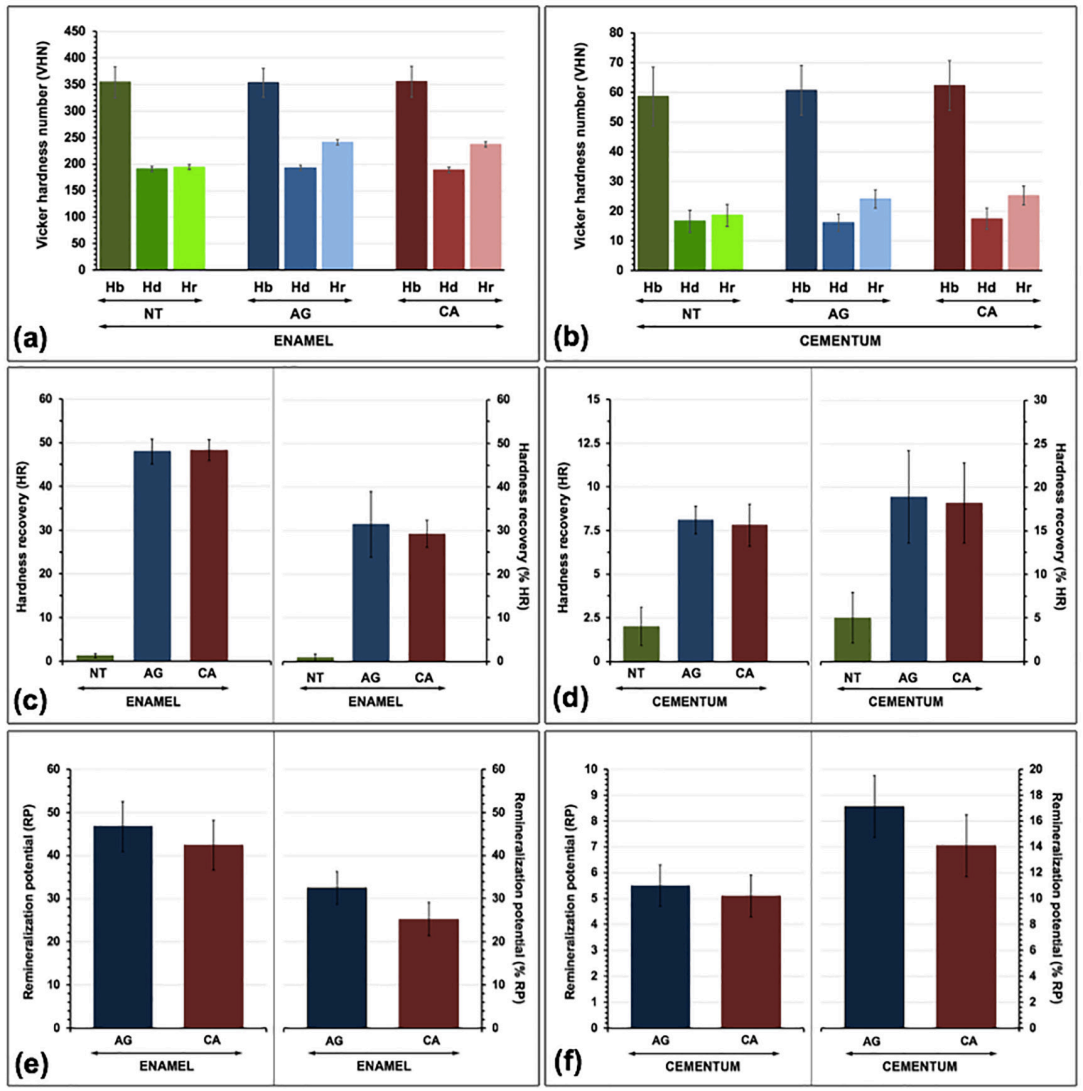
The mean, standard deviation of enamel (a) and cementum (b) at baseline hardness (Hb), hardness after the artificial formation of demineralization (Hd), hardness after application of remineralizing agent (Hr) with either apacider gel (AG), or Casein phosphopeptide-amorphous calcium fluoride phosphate (CA), and calculated for hardness recovery (HR), percentage of hardness recovery (%HR) (c, d), as well as remineralization potential (RP), and percentage of remineralization potential (%RP) (e, f).

Seventy-five extracted human molars throughout the suggestion of surgical extraction, without congenital anomaly, dental fluorosis, abrasion, crack line, dental caries, and white spot lesions were selected for the investigation. The patients and/or guardians were notified and endorsed in the consent beforehand. All teeth were immersed in the 0.1% thymol (M-Dent, Bangkok, Thailand) until used in the experiment. The teeth were precisely segmented with a sectioning apparatus (Isomet-4000, Buehler, IL, USA) at 0.8 mm beyond the CEJ, resulting in three separated portions [Fig. 2a-(1)]. The crown-CEJ-root (CCR) part was detached and substituted with the lithium disilicate (LS2, Emax CAD, Ivoclar-Vivadent, Schaan, Liechtenstein) glass-ceramic disk [Fig. 2a-(2)]. The LS2 glass-ceramic blank was shaped in an analogous conFiguration as CCR with 1.6 mm thickness and further sintered in the ceramic furnace (Programat P68, Ivoclar-Vivadent), consistent with the manufacturer firing schedule at 820 °C for 30 minutes and 840 °C for 30 minutes. The LS2 glass ceramic disk was joined to the crown (C) and root (R) parts with resin adhesive (Super-Bond, Sun Medical, Shiga, Japan) [Fig. 2a-(3)] with exactly 25 microns (µ) thickness, controlled by a digital vernier caliper (Mitutoyo, Aurora, IL, USA), and left for 10 minutes to completely auto-polymerize. The specimen was submersed in the resin blank (Unifast-Trad, GC, Tokyo, Japan) with one unprotected side for further investigation [Fig. 2a-(4)]. The exposed side was flattened for 4x4 mm2 using silicon carbide abrasive paper up to #4000 in a grinding machine (Ecomet-3, Buehler) [Fig. 2a-(5)]. The nail paint (Revlon, New York, NY, USA) was used to coat all exposed surfaces, except for the 4x4 mm2 region and then immersed in 37°C deionized water (DW) for 24 hours.

-Establishment of simulated caries 

The synthetic carious lesion was induced by a demineralized gel (DG) that is composed of 20 g/L of Carbopol-907 (BF-Goodrich, Cleveland, OH, USA), 500 mg/L of HA, 0.1% of lactic acid, and adjusted-pH to 5.0 by sodium-hydroxide. The tooth specimen was immersed in the DG for 16 hours at a humid ambiance and subsequently rinsed with DW to eliminate the DG and generate a 4x4 mm2 area of demineralization of enamel and cementum (25).

-Generating remineralization process

The specimens were unintentionally classified into 3 groups (n=25) based on different remineralized products ( Table 1) to be treated with either apacider gel (AG, Biomaterial research, KKU) or CPP-ACFP paste (CA, MI Paste Plus, GC), whilst one group was left no treatment (NT) by immersing in DW to assign for control. The tested product was wiped over the 4x4 mm2 demineralized area of enamel and cementum for 4 minutes and then soaked in a freshly prepared DW. The product was applied two times daily, at 12-hour intervals, for 30 days, and maintained in the DW at 37ºC with 95% relative humidity condition.

-Assessment of surface microhardness

The microhardness was measured along the location of 40 µ beyond the junction of adhesive resin to enamel and cementum for three phases: before demineralization (Bd), after demineralization (Ad), and after remineralization (Ar). Each phase was unintentionally assessed for Vickers hardness number (VHN) at five locations, at 100 µ apart (Fig. 2b). The Vickers diamond indenter was used to indent on enamel with 100 grams-force and on cementum with 25 grams-force for 15 seconds in the hardness tester (Digital FM-800, Future-tech, Tokyo, Japan) (Fig. 2c). The pyramidal shape indentation was measured in a diagonal direction (D1, D2) (Fig. 2d) and quantified for hardness before demineralization (Hb), after demineralization (Hd), and after remineralization (Hr) (17). The percentage of hardness recovery (%HR) and remineralization potential (%RP) were calculated per Equation (2) and (3), (Figs. 3,4).

Which: Hr* denotes hardness once the application of remineralization to NT.

-Microstructural evaluation

The specimens (15/group) were longitudinally segmented at 200 µ thick, rinsed with DW, and evaluated with a polarized light microscope (PLM, Eclipse-80i, Nikon, Kanagawa, Japan) at 10x magnification. The microscopies of normal intact as well as demineralized enamel and cementum were used as references for comparison with other examined groups. The alterations of enamel and cementum surface adjacent to the ceramic material were compared among the Bd, Ad, and Ad phases. The specimens (10/group) were coated with gold in a sputtering apparatus (Emitech-K500X, Quorum, Asford, UK) and observed for apparent characteristics using the scanning electron microscope (SEM, S-3000N, Hitachi, Tokyo, Japan) at x2K magnification.

-Statistics analysis

The VHN data were scrutinized using the SPSS-V20 program (IBM, Armonk, NY, USA). The analysis of variance (ANOVA) was employed to validate the statistically significant difference upon different remineralized materials at distinctive stages of experimentation comprising Hb, Hd, Hr, and HR, %HR, RP, and %RP for both enamel and cementum. Bonferroni multiple comparisons were introduced to validate for post-hoc significant differences among groups (α=0.05). The PLM and SEM microscopies were descriptively assessed.

## Results

The capability in remineralization either on enamel or cementum neighboring the ceramic restoration considered from the Vickers surface hardness were described in terms of mean and standard deviation (sd) of Hb, Hd, Hr, HR, %HR, RP, and %RP for each group ( Table 2, Fig. 5a-f). The ANOVA and Bonferroni multiple comparisons confirmed no significant difference in Hb amongst the experimental groups on either enamel or cementum (*p*>0.05) ( Table 3a,  Table 4a). The means Hd for all experimental groups were decreased compared to the means Hb for either enamel of cementum (Fig. 5a,b). Nonetheless, no significantly different Hd was exhibited among groups (*p*>0.05) ( Table 3b, Table 4b). After the process of remineralization was initiated, the means Hr for all groups were considerably improved compared with Hd (*p*<0.05), excluding the NT groups (*p*>0.05) (Fig. 5a, b and  Table 3c,d). ANOVA signified significant differences in the means Hr midst the examined groups either on enamel or cementum (*p*<0.05) (Fig. 5a,b,  Table 3c). The means Hr midst the examined groups signified significant differences, except between AG and CA for both enamel and cementum ( Table 4c). ANOVA suggested significant differences in the means HR and %HR amid the examined groups either on enamel or cementum (*p*<0.05) (Fig. 5c,d,  Table 3d,e). Post-hoc Bonferroni test designated significant differences of the means HR and %HR amid the examined groups excluding AG and CA for both enamel and cementum (*p*<0.05) ( Table 4d,e). The greatest %HR was noticed for the groups treated by AG, tailed CA, and NT, correspondingly. ANOVA indicated substantial differences in means RP and %RP midst the examined groups either on enamel or cementum (*p*<0.05) (Fig. 5e,f,  Table 3f,g). Bonferroni tests signified substantial differences in means RP and %RP amongst groups (*p*<0.05) were indicated, except for AG and CA for both enamel and cementum ( Table 4f,g). The use of AG and CA indicated significantly proficient capability in remineralization for the demineralized enamel and cementum (*p*<0.05) compared to NT. However, the remineralized proficiency of AG seems to be greater than CA for both enamel and cementum.

**Figure 6:**
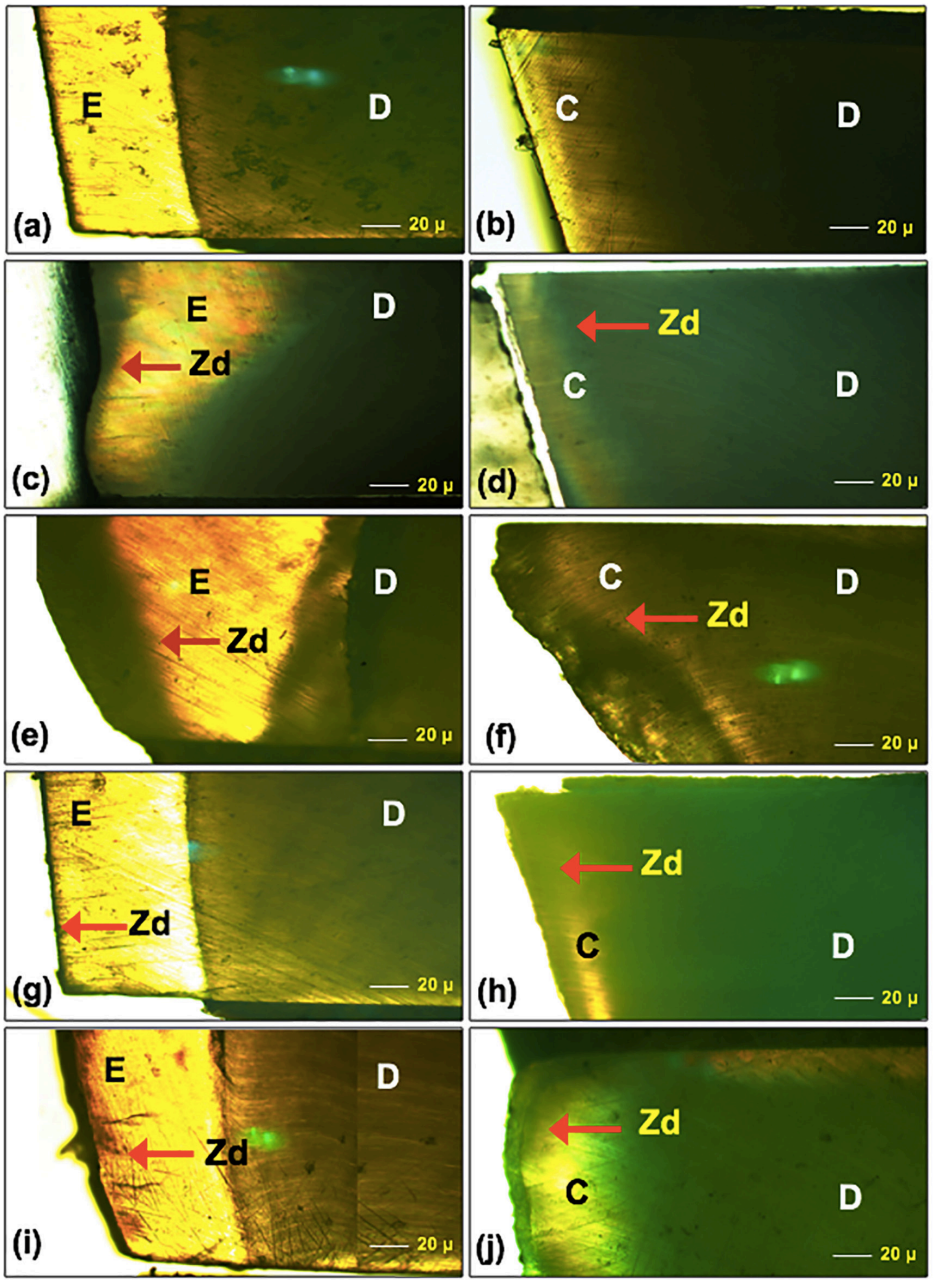
Polarized light micrograph (PLM) at x10 of enamel (a,c,e,e,g), and cementum (b,d,f,h,i) before artificial formation of demineralization (a,b), after demineralized (c,d), and after applied with remineralization agent either apacider gel (AG) (g,h) or casein phosphopeptide-amorphous calcium fluoride phosphate (CA) (i,j), compared to no treatment (NT) (e,f) as a control group. Demineralized lesion zone (Zd) indicated a difference of invasion from enamel (E) and cementum (C) through dentine (D).

The appearance of the carious area together with the progression of the remineralized process was explained by the PLM microscopies (Fig. 6) as compared to the PLM of an intact tooth with no evidence of caries (Fig. 6a,b). A visible ghostly region and enhancing depth of lesion were noticed with the PLM-specimen with caries (Fig. 6c, d). After the remineralization process was employed, the diminishing in lesion depth for each tested material was denoted (Fig. 6g-j), excluding the control group (NT) revealing an advancing lesion depth (Fig. 6e,f) compared with the depth of demineralized area (Fig. 6c,d). The experiment suggested that all remineralization materials were capable of engender remineralization of the carious lesion on both enamel and cementum. The decrease in carious lesions for both the AG and CA groups was superior to the NT groups. Still, the decrease in the carious lesion depth of the AG group perhaps implied a marginal superiority over the CA-treated group. Increasing in the carious lesion depth for the NT group was noticed, signifying no remineralization was established.

**Figure 7:**
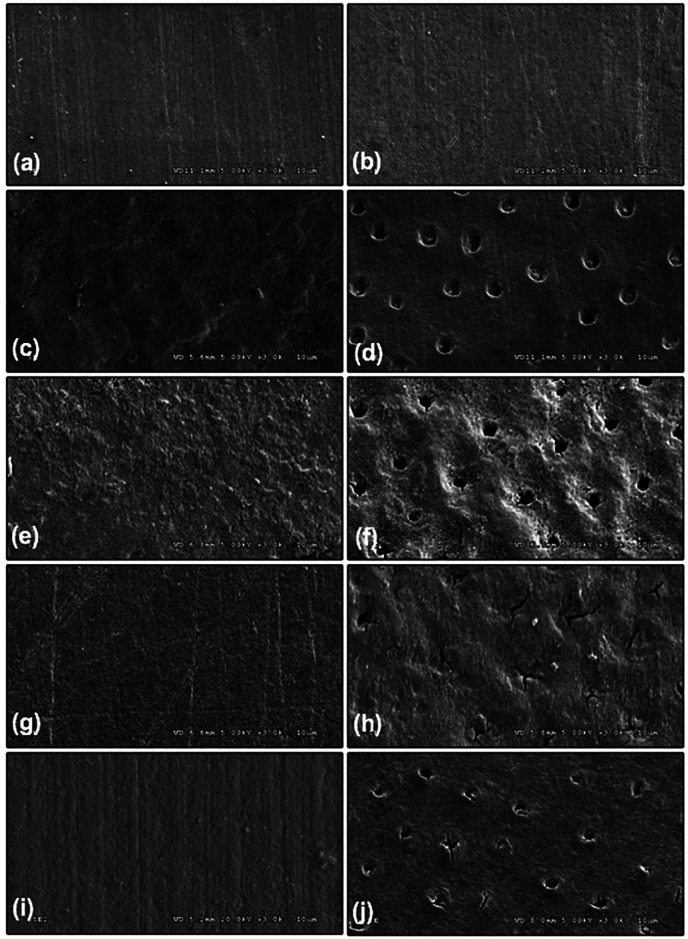
Scanning electron microscopy (SEM at x2K) of enamel (a,c,e,e,g), and cementum (b,d,f,h,i) before artificial formation of demineralization (a,b), after demineralized (c,d), and after applied with remineralization agent either apacider gel (AG) (g,h) or casein phosphopeptide-amorphous calcium fluoride phosphate (CA) (i,j), compared to no treatment (NT) (e,f) as a control group.

The SEM micrographs of intact enamel and cementum indicated generalized smooth surface architecture (Fig. 7a,b). Widespread apparent irregularities of demineralized enamel (Fig. 7c) and comprehensive indiscretions of demineralized cementum containing open tubules (Fig. 7d) were expressed following the process of demineralization. The untreated surfaces of demineralized enamel (Fig. 7e) and cementum (Fig. 7f) were not changed in surface characteristics. Whilst the surface characteristics of enamel and cementum exhibited glossier area once the AG (Fig. 7g,h) and CA (Fig. 7i,j) were treated to the demineralized surfaces for 30 days, in comparison to un-treated demineralized surfaces (Fig. 7e,f). The micrograph revealed the AG particles accumulation in the cementum tubules, together with a widespread decrease of apparent irregularities (Fig. 7h). Contrarily, several CA particles were partly precipitated in the tubules of cementum with a slight reduction in surface irregularities (Fig. 7j). The SEM micrograph for the NT groups demonstrated numerous sponginess and apparent roughness, classically associated to no establishing remineralization process (Fig. 7e,f). The SEM micrographs of the AG groups displayed a superior consistent manifestation, especially on the enamel representing the formation of a process of remineralization on the caries lesion. The SEM micrograph for the CA groups exposed a minimal irregular with microporosities, especially on the cementum that implied formulation of remineralization of the demineralized region, deprived of complete remineralization process (Fig. 7j) that indicated inadequacy of remineralization capacity of CA through the entire caries lesion.

## Discussion

A completely united connection of restoration to prepared abutment tooth is an essential goal that has scarcely succeeded. Certainly, imperfection at the restoration-tooth connection and microleakage at the restoration-tooth interfaces are constantly existent and provoke bacteriological aggregation causing dental caries. This investigation evaluated the abilities of AG and CA in remineralization at the cavosurface region of either enamel or cementum adjacent to the restorative ceramic. The study signified a significant difference in remineralization efficiency among groups, as specified by the rejection of the null hypothesis. This infers that both AG and CA can engender remineralization on the artificially carious lesion of enamel and cementum. Nevertheless, the remineralization potential between AG and CP was statistically comparable. The ability to remineralization on demineralized enamel and cementum was verified by the SEM. The particles of either AG or CA accumulated on the demineralized lesion of enamel and cementum in comparison with the NT. Particularly, the PLM microscopies elucidated a reduction in carious lesion depth once the demineralized enamel or cementum was applied with either AG or CA in comparison with the NT group. Nevertheless, the AG seems to signify a slightly superior ability in remineralization on either demineralized enamel or cementum than CA, but not statistical significance, as evidence from SEM indicated more particles of AG than CA deposition on the area of remineralized surface of enamel as well in the tubule of cementum. Also, the PLM seems to denote of better decrease in lesion depth of either demineralized enamel or cementum upon the application of AG than the application of CA. This undoubtedly signified that the ion transportation mechanism occurred in the remineralization process (10). The matrix protein comprising approximately 1% of the organic portion in matured enamel probably remained once demineralization and functioned as the core skeleton for ions transmission and deposition in the nano-gaps of the inter-prismatic area, as described by previous studies (15,24). The inter-prismatic matrix proteins were perhaps able to seize the mineral contents and permitted mineral infiltration along the crystalline structure (10,24). It also correlated with the isoionic and isomorphic exchanging process in crystalline enamel that happened in the transmission of Ca2+ and PO43- ions through the inter-prismatic lattices and turned into HA crystals (10). These proteins possibly operated as a skeleton for ion-exchanging of AG and CA (24). This is related to the proficiencies of AG and CA to exhibit the process of remineralization (18). The AG seems to exhibit greater ability in the remineralized process than CA, which is perhaps correlated to the nano-particle of AG, which is susceptible to the HA structure of the tooth. The AG is capable of increasing the permeation of its crystals across the inter-prismatic enamel structure and results in the establishment of the HA crystals (10). The CA might confront difficulty in forming a crystalline to endorse surface remineralization, but could only substitute the mineral missing through the forming of fluorapatite (10).

The surface of the demineralized enamel was substantially irregular and somewhat spongy. This appearance assisted the AG to infiltrate into the inter-prismatic lattices during the precipitation procedure of the material. It also fascinated a great quantity of Ca2+ and PO43- from the saturated mixture surrounding the surface to fill into the empty locations of the crystal lattice (11,28). The demineralized cementum showed the exposed tubules on the surface. Numerous quantities of inorganic material were accumulated in the tubules to facilitate the remineralization process (2,3). The remineralized process of cementum once applying AG or CA is perhaps correlated to the mineral exchanging process between cementum and neighboring condition as established in enamel. Nevertheless, the process of remineralization of cementum is less efficient than enamel as supported by the PLM microscopies, which is perhaps associated with the absence of protein skeleton in cementum to encourage the remineralization procedure (11,19). Yet, the greater proficiency in cementum remineralization of AG than CP is possibly related to the nano-size of particles that can accomplish inter-digitation to cementum structure (19). The DW was used in this study instead of artificial saliva to eradicate the perplexing factors that might happen from other substances, for example, the amino acids in saliva that may influence on natural remaining proteins in the tooth structure (21). Furthermore, the mineral contents in artificial saliva could generate confusing factors through remineralization, since the deproteinated enamel marks a significant decrease in enamel characteristics (15,19).

This study displayed that restricted remineralization of artificial carious lesions of enamel was possible because of Ca2+ and PO43- ions insufficiency while artificial caries development (6,16). The AG was capable of providing Ca2+ and PO43- ions that are concurrently dispersed into sub-surface areas for repairing crystal structure (13). Likewise, Sodata *et al*. indicated consistently remineralized artificial caries lesions once applied with the remineralization material (30). The porous appearance as well as lesion depth of caries illustrated a significant function for inorganic distribution. The bigger porosities usually convince greater inorganic installation, nevertheless, the worse lesion depth with deeper areas probably generates much more difficulties for inorganic captivation (20). The study can be described for the AG groups which possessed tiny molecules of Ca2+ and PO43- ions might be engaged through the deep porous appearance of the tooth and accumulated considerably farther quantities of inorganic ions than CA as revealed from the higher surface microhardness (12,26).

The groups treated with AG demonstrated fairly smoothness of the enamel surface. This was coherent with some experiments that explained the capability of apacider on enamel remineralization exhibiting better harmonized and smooth surfaces (22,29,30). Furthermore, the geomorphology of specimens after attacking with AG showed a homogeneous smooth enamel compared to the untreated specimens (13). Likewise, this study supported that the tooth treated with AG demonstrated a further homogeneous and glossier surface of enamel than that treated with CA (20). This might be associated with the partly demineralized crystal structure inside the carious lesion performing like a “nucleator” for establishing a newly developed crystalline structure in the constitution of the remineralization process by mineral substitution in the partly demineralized zones of the enamel caries lesion (12). The anti-caries mechanism of AG is associated with the integration of calcium and phosphate nano-complexity on tooth structure and acts as a pool of Ca2+ and PO43- ions which are continuously released farther to the internal part of porous enamel, with extremely accumulated at the sub-surface regions. The greater remineralization capacity of AG, compared to CA, was addressed in this study as confirmation with the SEM photomicroscopy that AG-treated specimens revealed a consistently smooth surface beside a tiny amount of remaining porosity, while the CA-treated specimens exhibited an irregular manifestation and minimal remaining porosity. These characteristics were probably associated with the analogous appearances of AG to the conFiguration of enamel which perhaps engendered the encouragement in the remineralization process and provoked the formation of a constant HA structural complexity on the demineralized enamel once the gel was applied (30). On the other hand, the CA helped establish intraoral fluoride reservoirs due to the formation of the CaF2. This structural complexity incorporates the synthetic HA by directly attaching to the enamel crystalline structure. Therefore, the surface hardness between the remineralized- and healthy enamel is quite equivalent (17). Contrariwise, the enamel that was remineralized with CA required Ca2+ and PO43- to generate fluorapatite, which limited ions offered in the saliva, therefore the remineralization might not be entirely achieved (16,23,30).

The PLM micrographs precisely measured the depth of demineralized lesions in various experimental groups. This accurateness is necessary for the correct assessment of such treatment agents that might not be prohibited by the demineralized process, though, it might decrease the range of the exaggerated tooth surface amongst the amount of mineral damage and the lesion depth (8). The PLMs of the early carious lesion after the therapeutic agent was used displayed a condensing strip beneath the external surface of the enamel. For the specimen receiving AG, a smaller band defined from color intensity and the width of the band underneath the external surface of enamel was shown, compared to CA, and NT groups, indicating the higher capability of AG in remineralization. The PLMs proved that the AG-treated groups were capable of decreasing the depth of demineralized enamel compared to other groups. This was possibly related to the size of the major component of AG which comprised of Ca2+ and PO43- ions. The particle sizes of Ca2+ [Ø=1.98 angstrom (Å)] and the PO43- (Ø=2.30 Å) ions are smaller than F- ion (Ø=2.64 Å), thus the AG is better remineralization than CA. The nano-crystalline conFiguration of AG is easily gathered in the caries lesions, and ultimately decreases the depth of lesion. The investigation is coherent with other studies that the apacider-treated group revealed less demineralized depths than other remineralized materials (22,29,30).

The experiment signified that the %RP and the %HR of AG were equivalent to CA. Nonetheless, the evidence from SEM and PLM revealed that AG had more potential in remineralization than CA, which was correspondingly consistent with other studies (22,29,30). The lesser particle size of AG probably helped the particles better penetration throughout the demineralized enamel and continuously filled in the porous lesion of the caries better than CA. The SEM photomicrographs demonstrated that AG was efficient in finding a harmonized apatite deposition, which resembled former studies (29,30). Furthermore, this experiment advised that both AG and CA were capable of raising the microhardness of carious lesions. The remineralization proficiency of AG relates to the acid surrounding condition that escalated the solubility of AG and therefore intensified the accumulation of AG on carious enamel (27).

The limitations of this experiment associated with the study design that limited by mimicking salivary pellicles and biofilm as existing in the oral cavity. Likewise, each tooth sample was different due to the maturity of donors as well as the exposed experience in oral situations. This probably caused an aberration in the reactions with the acid-challenged conditions. Also, the duration of the demineralization-remineralization process in this study was briefer than the in-vivo circumstance (20). Further clinical investigations are advised to validate the remineralized capability of AG. However, the allegation of this study was decisive employing the exploration of the novel remineralized material to provide well-meaning information for dentists to select for their proficient dental practice.

## Conclusions

The use of remineralized material to battle initial carious lesions surrounding the cavosurface margin of restorations throughout the process of remineralization is a somewhat innovative method in restorative dentistry. This in-vitro investigation afforded the remineralized capability of AG equivalent to CA for carious enamel and cementum remineralization. The aspect of early carious lesion as the principal factor for failure of restorations, both AG and CA signified efficient remineralization proficiencies for both enamel and cementum neighboring the cavosurface margin of ceramic restoration, thus considering as an effective method to inhibit caries progression. The AG provided strong evidence of surface remineralization capability slightly better than CA for both enamel and cementum carious lesions.

Clinical implications

Although dentists are tremendously capable of delivering the finest ceramic restorations for their patients, marginal discrepancies in the restorations still occur, which challenge the competence of materials like AG to Fight early carious lesions. It is undoubtedly suggested for medically compromised patients who might otherwise possess difficulties in oral hygiene care. The use of AG twice a day significantly provides remineralization potential for demineralized enamel or cementum. The ability of AG suggested an auspicious paradigm in a preventive aspect of contemporary fixed prosthodontic treatment to minimize the possibility of decalcification of either enamel or cementum surrounding the restorative margin in addition to increasing longstanding treatment success. The finding signified a new approach in conservative dentistry and established a modern preventive philosophy in contemporary fixed prosthodontic treatment.

## Figures and Tables

**Table 1:** Materials, company, and compositions of materials used in this study.

Materials	Company	Composition
Apacider gel (AG)	Biomaterial research, KKU, Khon Kaen, Thailand	Apacider^®^ AW (Sangi, Tokyo, Japan) 1.25 g, Deionized water 12.5 ml, Sodium carboxymethyl cellulose (SCMC) 416 mg
Casein phosphopeptide-amorphous calcium fluoride phosphate (CA)	MI Paste Plus, GC Int, Tokyo, Japan	Casein phosphopeptide-amorphous calcium phosphate plus 900 ppm (0.2% w/w) sodium fluoride
Demineralized gel (DG)	Biomaterial research, KKU Khon Kaen, Thailand	Carbopol-907 (BF Goodrich, Cleveland, OH, USA) 20 g/liter, 500 mg/liter Hydroxy apatite, Polyacrylic acid 0.2%, and Lactic acid 0.1%, and Sodium hydroxide for adjusting pH to 4.4

**Table 2:** Mean, standard deviation (sd) of hardness before demineralization (Hb), hardness after demineralization Hd), hardness after remineralization (Hr), hardness recovery, (HR) percentage of hardness recovery (%HR), remineralization potential (RP), and percentage of remineralization potential (%RP) of enamel and cementum for groups of no treatment (NT), apacider gel (AG), and casein phosphopeptide-amorphous calcium fluoride phosphate (CA).

Enamel							
Group	Hb	Hd	Hr	HR	%HR	RP	%RP
	Mean±sd	Mean±sd	Mean±sd	Mean±sd	Mean±sd	Mean±sd	Mean±sd
NT	354.6±29.1^a^	191.4±5.1^b ^	194.6±4.9^c^	1.3±1.5^e^	0.8±0.9^g^	0.0	0.0
AG	353.6±27.1^a ^	193.4±5.1^b ^	241.4±4.9^d^	48.0±2.9^f^	31.4±7.5^h^	46.7±5.8^k^	32.5±3.8^m^
CA	355.6±29.1^a ^	188.8±5.1^b^	237.1±4.9^d^	48.3±2.4^f^	29.2±3.1^h^	42.4±5.8^k^	25.2±3.8^m^
Cementum							
Group	Hb	Hd	Hr	HR	%HR	RP	%RP
	Mean±sd	Mean±sd	Mean±sd	Mean±sd	Mean±sd	Mean±sd	Mean±sd
NT	58.6±9.9^n^	16.6±3.7^p ^	18.6±3.8^q^	2.0±1.1^s^	5.0±2.9^u^	0.0	0.0
AG	60.7±8.4^n ^	16.1±2.9^p ^	24.1±3.1^r^	8.1±0.8^t^	18.9±5.3^v^	5.5±0.8^w^	17.1±2.4^x^
CA	62.28±8.4^n ^	17.5±3.5^p^	25.3±3.2^r^	7.8±1.2^t^	18.2±4.6^v^	5.1±0.8^w^	14.1±2.4^x^

Different superscript letters in the same column denoted significant differences between groups (*p*<0.05).

**Table 3:** An analysis of variance (ANOVA) of hardness before demineralization (Hb), hardness after demineralization (Hd), hardness after remineralization (Hr), hardness recovery (HR), percentage of hardness recovery (%HR), remineralization potential (RP), and percentage of remineralization potential (%RP) of enamel and cementum among groups of treatment.

Enamel						Cementum				
a. One-way ANOVA of Hb										
Source	SS	df	MS	F	p	SS	df	MS	F	p
Group	52.40	2	26.20	.034	.966	158.72	2	79.36	1.08	.346
Error	55037.52	72	764.41			5306.26	72	73.70		
Total	55089.92				74	5464.98	74			
b. One-way ANOVA of Hd										
Source	SS	df	MS	F	p	SS	df	MS	F	p
Group	350.59	2	175.29	.270	.764	27.39	2	13.70	1.17	.316
Error	46757.41	72	649.41			841.18	72	11.68		
Total	47108.00	74				868.57	74			
c. One-way ANOVA of Hr										
Source	SS	df	MS	F	p	SS	df	MS	F	p
Group	33357.31	2	16678.6	22.86	.001	637.25	2	318.62	27.78	.001
Error	44703.86	72	620.89			825.70	72	11.47		
Total	78061.17	74				1462.95	74			
d. One-way ANOVA of HR										
Source	SS	df	MS	F	p	SS	df	MS	F	p
Group	36536.71	2	18268.4	3344.3	.001	576.43	2	228.21	270.1	.001
Error	393.30	72	5.46			76.84	72	1.07		
Total	36930.01	74				653.27	74			
e. One-way ANOVA of %HR										
Source	SS	df	MS	F	p	SS	df	MS	F	p
Group	14575.45	2	7287.72	325.6	.001	3032.76	2	1516.4	78.42	.001
Error	1611.56	72	22.83			1392.09	72	19.34		
Total	16187.01	74				4424.85	74			
f. One-way ANOVA of RP										
Source	SS	df	MS	F	p	SS	df	MS	F	p
Group	33357.31	2	16678.7	19.60	.001	637.25	2	318.62	18.08	.001
Error	61265.68	72	850.91			1268.96	72	17.62		
Total	94622.99	74				1906.21	74			
g. One-way ANOVA of %RP										
Source	SS	df	MS	F	p	SS	df	MS	F	p
Group	14560.80	2	7280.4	19.23	.001	4180.39	2	2090.2	14.20	.001
Error	27259.51	72	378.6			10594.81	72	147.15		
Total	41820.31	74				14775.20	74			

Abbreviations: df: degree of freedom, F: F-ratio, MS: mean square, p: *p*-value, SS: sum of squares.

**Table 4:** Post hoc Bonferroni multiple comparisons of hardness before demineralization (Hb), hardness after demineralization (Hd), hardness after remineralization (Hr), hardness recovery (HR), percentage of hardness recovery (%HR), remineralization potential (RP), percentage of remineralization potential (%RP) of enamel and cementum among groups of no treatment (NT), apacider gel (AG), and casein phosphopeptide-amorphous calcium fluoride phosphate (CA).

a. Bonferroni multiple comparisons of Hb							
Enamel	NT	AG	CA	Cementum	NT	AG	CA
NT	1.000	1.000	1.000	NT	1.000	1.000	0.446
AG		1.000	1.000	AG		1.000	1.000
CA			1.000	CA			1.000
b. Bonferroni multiple comparisons of Hd							
Enamel	NT	AG	CA	Cementum	NT	AG	CA
NT	1.000	1.000	1.000	NT	1.000	1.000	0.976
AG		1.000	1.000	AG		1.000	0.409
CA			1.000	CA			1.000
c. Bonferroni multiple comparisons of Hr							
Enamel	NT	AG	CA	Cementum	NT	AG	CA
NT	1.000	0.001	0.001	NT	1.000	0.001	0.001
AG		1.000	1.000	AG		1.000	0.851
CA			1.000	CA			1.000
d. Bonferroni multiple comparisons of HR							
Enamel	NT	AG	CA	Cementum	NT	AG	CA
NT	1.000	0.001^*^	0.001^*^	NT	1.000	0.001^*^	0.001^*^
AG		1.000	1.000	AG		1.000	0.713
CA			1.000	CA			1.000
e. Bonferroni multiple comparisons of %HR							
Enamel	NT	AG	CA	Cementum	NT	AG	CA
NT	1.000	0.001^*^	0.001^*^	NT	1.000	0.001^*^	0.001^*^
AG		1.000	0.285	AG		1.000	1.000
CA			1.000	CA			1.000
f. Bonferroni multiple comparisons of RP							
Enamel	NT	AG	CA	Cementum	NT	AG	CA
NT	1.000	0.001^*^	0.001^*^	NT	1.000	0.001^*^	0.001^*^
AG		1.000	1.000	AG		1.000	1.000
CA			1.000	CA			1.000
g. Bonferroni multiple comparisons of %RP							
Enamel	NT	AG	CA	Cementum	NT	AG	CA
NT	1.000	0.001^*^	0.001^*^	NT	1.000	0.001^*^	0.001^*^
AG		1.000	0.563	AG		1.000	1.000
CA			1.000	CA			1.000

NB: * Significance at *p*<0.05

## Data Availability

The datasets used and/or analyzed during the current study are available from the corresponding author.
